# High Level Expression and Purification of the Clinically Active Antimicrobial Peptide P-113 in *Escherichia coli*

**DOI:** 10.3390/molecules23040800

**Published:** 2018-03-30

**Authors:** Kuang-Ting Cheng, Chih-Lung Wu, Bak-Sau Yip, Hui-Yuan Yu, Hsi-Tsung Cheng, Ya-Han Chih, Jya-Wei Cheng

**Affiliations:** 1 Institute of Biotechnology and Department of Medical Science, National Tsing Hua University, Hsinchu 300, Taiwan; s8888222211@gmail.com (K.-T.C.); oops801011@gmail.com (C.-L.W.); neuron.hch@gmail.com (B.-S.Y.); gwalt1103@gmail.com (H.-Y.Y.); habercheng@gmail.com (H.-T.C.); chih80517@gmail.com (Y.-H.C.); 2 Department of Neurology, National Taiwan University Hospital, Hsinchu 300, Taiwan

**Keywords:** P-113, hG31P, expression, purification, NMR

## Abstract

P-113, which was originally derived from the human saliva protein histatin 5, is a histidine-rich antimicrobial peptide with the sequence AKRHHGYKRKFH. P-113 is currently undergoing phase II clinical trial as a pharmaceutical agent to fight against fungal infections in HIV patients with oral candidiasis. Previously, we developed a new procedure for the high-yield expression and purification of hG31P, an analogue and antagonist of human CXCL8. Moreover, we have successfully removed lipopolysaccharide (LPS, endotoxin) associated with hG31P in the expression with *Escherichia coli*. In this paper, we have used hG31P as a novel fusion protein for the expression and purification of P-113. The purity of the expressed P-113 is more than 95% and the yield is 4 mg P-113 per liter of *E. coli* cell culture in Luria-Bertani (LB) medium. The antimicrobial activity of the purified P-113 was tested. Furthermore, we used circular dichroism (CD) and nuclear magnetic resonance (NMR) spectroscopy to study the structural properties of P-113. Our results indicate that using hG31P as a fusion protein to obtain large quantities of P-113 is feasible and is easy to scale up for commercial production. An effective way of producing enough P-113 for future clinical studies is evident in this study.

## 1. Introduction

Antimicrobial peptides normally consist of 12 to 50 amino acids in length and fold into α-helices, β-sheets, extended, and looped peptides [1,2]. The main microbicidal mechanism of antimicrobial peptides to fight against bacteria and fungi is to incorporate and permeabilize the microbial membranes, thus causing the destruction of the microbes [3]. Because of this special mechanism, antimicrobial peptides may serve as promising therapeutic agents in the fight against antibiotic resistances [4].

While *Candida albicans* is naturally present in the mouth of many individuals, it can cause severe health problems to patients associated with immune deficiency diseases, such as AIDS, cancer, and diabetes. P-113, which is an antimicrobial peptide obtained from the saliva protein histatin 5, was shown to have excellent anti-*Candida* activities [5]. Recently, it was reported that P-113 is safe and effective in the clinical trials for HIV patients with oral candidiasis [6]. In addition, P-113 has been studied in humans with experimental gingivitis [7,8,9]. The results showed that P-113 reduces gingivitis, gingival bleeding, and plaque in humans, and it is safe. The advantages of P-113 over the current treatments for oral infectious diseases are obvious due to its safety profile and reduced risk of antibiotic resistance.

Antimicrobial peptides can be derived from chemical synthesis. However, the cost of synthesis has become a major impediment in developing antimicrobial peptides as pharmaceutical agents [10,11]. The biosynthesis of recombinant antimicrobial peptides provides an alternative method to prepare these peptides. Among the expression systems, *Escherichia coli* is most commonly used due to its easy DNA manipulation, high expression level, and less production time [11]. But, problems were found in recovering antimicrobial peptides from *E. coli* because of its susceptibility to proteolytic degradation, toxicity to host cells, and difficult purification procedures. Various fusion partners have been used to overcome these problems, including the small ubiquitin-like modifier (SUMO) protein [12], thioredoxin [13], baculoviral polyhedrin (Polh) [14], maltose-binding protein [15], green fluorescent protein (GFP) [16], the RepA protein [17], F4 fragment of PurF [18], intein [19], glutathione S-transferase [20], protein PaP3.30 [21], anion peptide [22], and calmodulin (CAM) [23]. Usually, the fusion partners must be cleaved after purification to obtain the desired biological activities of these antimicrobial peptides. However, the purification and cleavage methods can be time-consuming and expensive. Previously, we have established a new method for high-yield expression and purification of hG31P, an analogue and antagonist of human CXCL8 [24]. Moreover, we have successfully removed lipopolysaccharide (LPS, endotoxin) associated with hG31P from the expression in *Escherichia coli*. In this paper, we used hG31P as a novel fusion partner for expressing and purifying P-113. The purified P-113 was tested by various antimicrobial assays. We then used circular dichroism (CD) and nuclear magnetic resonance (NMR) to study the structural properties of the expressed P-113. The results of this study provide an effective protocol to generate large amounts of P-113 for future clinical investigations and to label with stable isotopes for nuclear magnetic resonance (NMR) structural studies and amino acid sequence modifications.

## 2. Results

### 2.1. Construction of the Recombinant Plasmid

**Figure 1:**
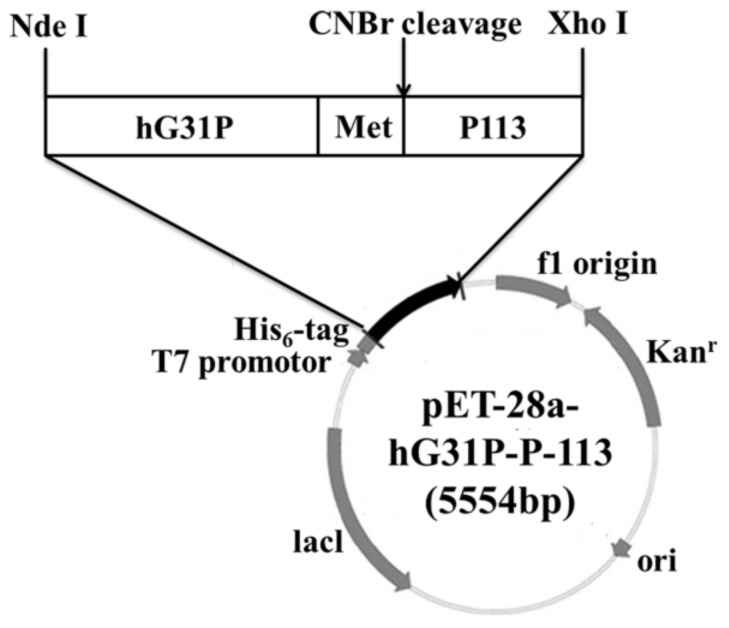
Gene map of the recombinant plasmid pET-28a-hG31P-P-113.

Previously, we have done cloning the hG31P DNA sequence with the pET-22b plasmid and expressed in *E. coli* (strain BL21(DE3)) [24]. Using the pET-22b-hG31P plasmid as a template, the gene encoding hG31P-P-113 was amplified and then subcloned into the expression vector pET-28a. DNA sequence analysis showed that the hG31P-P-113 sequence was correct. The final construction of the pET-28a-hG31P-P-113 plasmid contains an N-terminal His_6_-tag, the hG31P fusion protein, and a CNBr cleavage site for peptide release (Figure 1).

### 2.2. Expression, Extraction and Purification of hG31P-P-113

**Figure 2:**
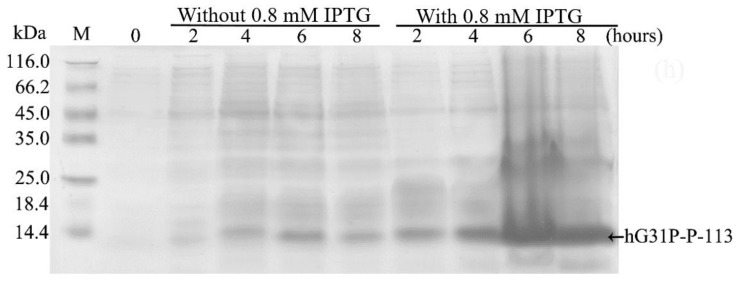
Sodium dodecyl sulfate (SDS)–polyacrylamide gel electrophoresis (PAGE) analysis of recombinant hG31P-P-113 expressed in *E. coli* BL21.

**Figure 3:**
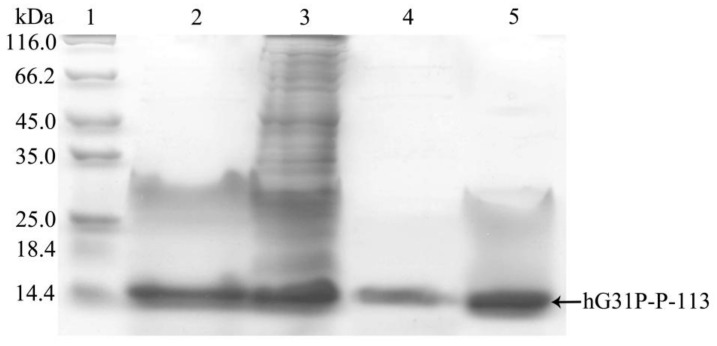
SDS-PAGE analysis of recombinant hG31P-P-113 expressed in *E. coli* at different steps of purification procedure. **Lane 1**: protein molecular weight markers; **lane 2**: supernatant of cell lysate; **lane 3**: pellet from cell lysate dissolved in 8 M urea; **lane 4**: supernatant after dialysis; **lane 5**: pellet after dialysis. Proteins were stained with Coomassie brilliant blue.

*E. coli* (BL21(DE3)) cells containing the pET-28a-hG31P-P-113 plasmid were successfully induced by IPTG, and using SDS–PAGE with Coomassie brilliant blue staining, the expression of the recombinant hG31P-P-113 protein was analyzed (Figure 2). Previously, we have developed a strategy to use high salt condition (i.e., 700 mM NaCl) and from the pellet in the cell lysate, extract more hG31P into the soluble form [24]. Subsequently, hG31P could be purified by heating the cell lysate to 70 °C for 10 min, and followed by rapid cooling to 0 °C [24]. The supernatant containing hG31P then underwent dialysis to remove the high salt concentration and was loaded onto a SP Sepharose column for purify further. Similarly, we were able to remove most of other bacterial proteins from the cell lysate and to obtain the hG31P-P-113 protein in the soluble form by combining the heating-cooling procedures under high salt concentration, (Figure 3).

**Figure 4:**
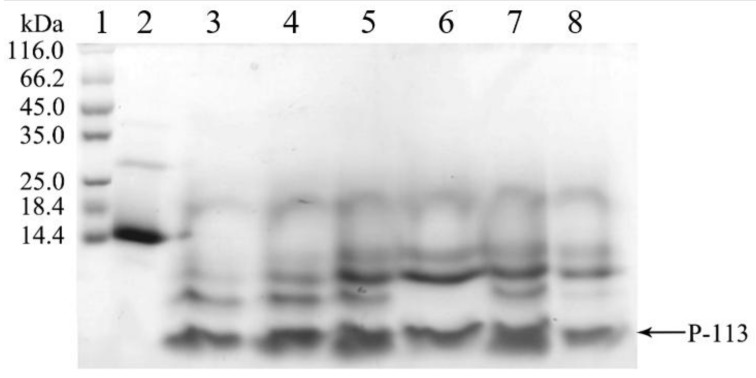
SDS-PAGE analysis of the cleavage of hG31P-P-113 at different times. **Lanes 1**: protein molecular weight markers; **Lane 2**–**8**: 0~6 h after CNBr cleavage. Proteins were stained with Coomassie brilliant blue.

Surprisingly, during the dialysis process to remove the high salt, the hG31P-P-113 protein began to precipitate. This problem turned out to be a favorable procedure to purify hG31P-P-113 because other unwanted proteins stayed in the soluble form during dialysis. The precipitant containing the hG31P-P-113 fusion protein was then obtained via centrifugation. The hG31P-P-113 protein was re-dissolved in 70% (*v*/*v*) formic acid and was cleaved by CNBr at room temperature for 6 h. The reaction was terminated by dilution (1:10) and the sample was dried under vacuum. The digestion by CNBr was verified by SDS-PAGE (Figure 4).

### 2.3. Quantification and Characterization of P-113

**Figure 5:**
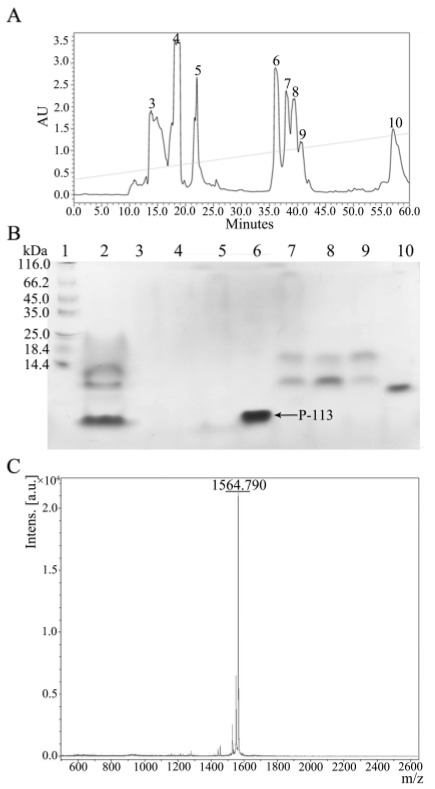
(**A**) Reversed-phase HPLC purification of hG31P-P-113 after cleavage by CNBr. (**B**) SDS-PAGE analysis of the HPLC fractions. P-113 peptide eluted out at 36 min. **Lane 1**: protein marker; **Lane 2**: mixture after CNBr cleavage; **Lanes 3**–**10**: peak collection of retention time from HPLC. Proteins were stained with Coomassie brilliant blue. (**C**) Mass analysis of fraction 6 from HPLC. The MW of the purified P-113 was found to be 1564.790 Da based on MALDI-TOF MS. The theoretical MW of P-113 was calculated to be 1564.870 Da.

Reversed-phase HPLC was used to purify the CNBr digested protein mixture with a gradient of water/acetonitrile containing 0.1% trifluoroacetic acid (TFA). HPLC chromatograms are given in Figure 5A. The retention time of P-113 was found to be at 36 min and was verified by SDS-PAGE (Figure 5B, **lane 6**). The molecular mass of the eluted P-113 was determined by MALDI-TOF MS. The molecular mass obtained from the corresponding spectrum was 1564.790 Da (Figure 5C), which is in line with the expected molecular mass of 1564.870 Da. The purity of the expressed P-113 is more than 95% and the yield is 4 mg P-113 per liter of *E. coli* cell culture in LB medium. The limulus amebocyte lysate (LAL) PYROCHROME test was used to detect the LPS (endotoxin) level. After HPLC purification, the LAL test was performed by using chromogenic endotoxin testing reagents (Pyrochrome^®^, Associates of Cape Cod, Inc., East Falmouth, MA, USA). The stock of control standard endotoxin (CSE) was diluted into 5, 4, 2, 1, 0.5 EU/mL as a standard curve. The LPS concentration of the expressed and purified P-113 was 1.4 EU/mg, which is appropriately equal to the LPS concentration of buffer.

### 2.4. Activity Assays

We have tested 10 *Candida* strains including drug resistant strains derived Dr. Hsiu-Jung Lo, National Health Research Institute, Miaoli, Taiwan, to confirm that the expressed P-113 still retained its antimicrobial activities (Table 1). The results showed that the antimicrobial activities of the expressed P-113 are comparable to that of the chemically synthesized P-113.

### 2.5. Characterization of the Expressed P-113 by CD and NMR

**Figure 6:**
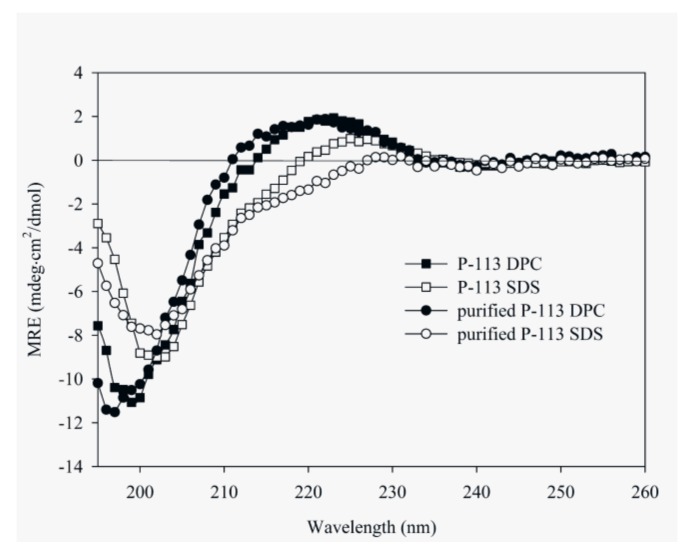
Circular dichroism (CD) spectra of 60 μM P-113 (chemically synthesized) and purified P-113 (expressed) in 25 mM sodium dodecyl sulfate (SDS) and DPC at 25 °C.

**Figure 7:**
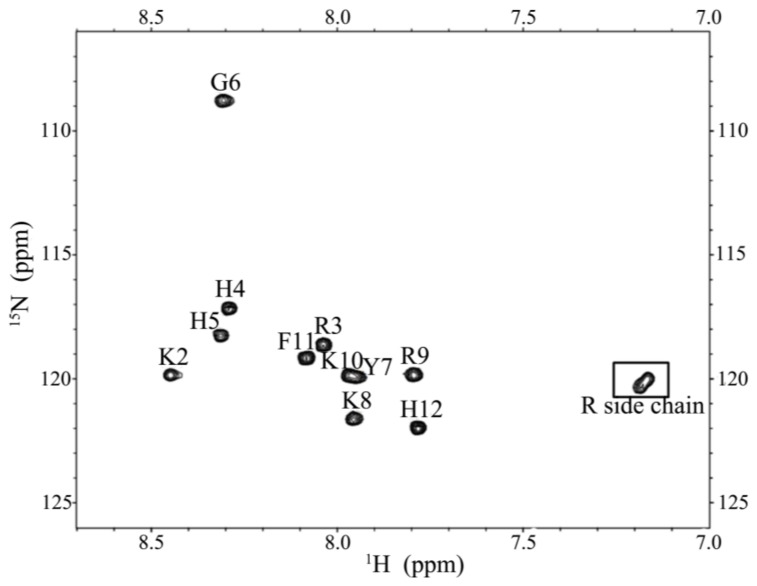
^1^H-^15^N HSQC of P-113 in SDS micelles. Cross-peaks are labeled according to the residue types and numbers.

CD spectroscopy was used in detecting the structural changes of P-113 while binding with sodium dodecyl sulfate (SDS) and dodecylphosphocholine (DPC) micelles (Figure 6). The result is comparable to the previous CD spectra of chemically synthesized P-113 [26]. In addition to the structural studies of P-113 by CD spectroscopy, we have successfully assigned the ^1^H and ^15^N resonance peaks of the expressed P-113 in SDS micelles. A well-resolved two-dimensional (2D) ^1^H-^15^N HSQC spectrum is shown in Figure 7. Previously, solution structure of P-113 (synthesized) in SDS micelles was determined by NMR spectroscopy [26]. The result indicated that P-113 had a helical segment from residues Ala1 to Tyr7 and adopted an amphipathic conformation. However, a divergent structure was found in the C-terminal domain. This is probably the reason why the CD spectra of P-113 recorded in SDS and DPC micelles do not show an apparent helical conformation. Nevertheless, in the present studies, both expressed P-113 and synthesized P-113 adopt similar conformation in SDS and DPC micelles.

## 3. Discussion

Antimicrobial peptides were discovered as a critical component in the host innate defense mechanism [4]. Moreover, because of a better understanding of the mechanisms of action and the rules that are governing the design and modification, many of the antimicrobial peptides are currently undergoing clinical development to fight against antibiotic resistances [27]. Among these peptides, P-113 is in phase II clinical trial for HIV patients with oral candidiasis [4]. However, the cost of chemical synthesis of P-113 has become a major obstacle to its development as a pharmaceutical agent. On the other hand, it is difficult to express cationic antimicrobial peptides in *E. coli* due to their toxicity to host cells and their sensitivity to the host intracellular proteolytic degradation [13]. A fusion protein expression system may overcome the toxicity and degradation problems. However, problems were still found to associate with inefficient cleavage of the fusion protein or the high cost of the cleavage methods that are used. In addition, the purification processes of the expressed protein/antimicrobial peptide can be costly and difficult to develop. Recently, we have developed a simple and effective protocol for high-yield expression and purification of recombinant hG31P, an analogue and antagonist of human CXCL8, in *E. coli* [24]. Herein, we have used hG31P as a fusion protein to successfully express hG31P-P-113 in *E. coli*. The expression level of hG31P-P-113 is very high, but a part of it is found in the pellets. To solve the above problems, we used high thermal stability of hG31P-P-113 to establish a simple yet effective procedure for high-yield purification of hG31P-P-113. By combining the heating-cooling procedures under high salt concentration, we were able to remove most of other bacterial proteins from the cell lysate and to extract more hG31P-P-113 in the soluble form. Subsequently, hG31P-P-113 was further purified by collecting the precipitated hG31P-P-113 in the dialysis process. The precipitated hG31P-P-113 was re-dissolved in 70% (*v*/*v*) formic acid and was cleaved by CNBr. The efficiency of the CNBr cleavage is very high and the cost is low. The cleaved products were then purified by HPLC to obtain the expressed P-113. The purity of the expressed P-113 is more than 95% and the yield is 4.0 mg P-113 per liter of *E. coli* cell culture in LB medium. This result is comparable or up to eight times better than other results that were reported using various fusion protein technologies [13,28,29,30]. We have also shown that the expressed P-113 adopts an ordered structure in SDS micelles, which is identical to the chemically synthesized sample. In addition, we have demonstrated that the ^15^N-labeled P-113 produced in this study may be used to understand the interactions between the antimicrobial peptide and model membranes/microbes, and to facilitate the design and development of more useful antimicrobial peptides. Most importantly, the purified P-113 retained its antimicrobial activities against various fungal strains.

In conclusion, we have successfully expressed and purified the clinically active antimicrobial peptide P-113 from *E. coli*. We have established a simple yet effective protocol for purifying P-113. A commercial scale production can be easily achieved with this protocol. The hG31P fusion technology can be also widely applied to obtain other antimicrobial peptides from *E coli*. Such studies are undergoing in our laboratories.

## 4. Materials and Methods

The expression vector pET-28a and host BL21 (DE3) were purchased from Novagen (Merck Millipore, Burlington, MA, USA). Restriction enzymes and T4 ligase were purchased from Thermo Scientific (Waltham, MA, USA). LB Broth medium was purchased from MDBio (Taipei, Taiwan), CNBr was purchased from Sigma-Aldrich (St. Louis, MO, USA). Sabouraud Dextrose (SD) Broth and SD agar were purchased from Becton, Dickinson and Company (Franklin Lakes, NJ, USA). ^15^N labeled ammonium chloride was purchased from Cambridge Isotope Laboratories, Inc. (Tewksbury, MA, USA). Other chemicals were from commercial sources.

### 4.1. Construction of the Expression Plasmid

A Met codon (a unique CNBr cleavage site) was introduced between hG31P and P113. The hG31P-P-113 DNA sequence was obtained by PCR and was located between NdeI and XhoI of the pET-28A plasmid. DNA sequences of hG31P-P-113 are listed as below:

5′ GAA TTC TGA CAT CAT ATG GGC TCT AAA GAA CTG CGT TGT CAA TGC ATT CGT ACT TAC TCT AAG CCA TTC CAC CCG AAG TTC ATC AAA GAA CTG CGT GTG ATT GAA TCT CCG CCA CAC TGC GCC AAT ACC GAA ATC ATT GTT AAA CTG AGC GAC GGT CGT GAA CTG TGT CTG GAC CCG AAA GAA AAT TGG GTA CAG CGT GTG GTG GAA AAA TTT CTG AAA CGT GCC GAA AAC TCT ATG GCG AAA CGT CAT CAT GGC TAC AAA CGT AAA TTC CAT TAA CTC GAG CGT 3′

Underlines are restriction sites of NdeI and XhoI, respectively.

After being digested with restriction endonucleases at 37 °C overnight, the gene fragment encoding hG31P-P-113 was purified by gel extraction and ligated into the pET-28a vector by T4 DNA ligase. The resulting vector, named pET-28a-hG31P-P-113, was collected after transformation into *E. coli* DH5α competent cells. The vector was checked on an agarose gel after double digestions with restriction endonucleases and was further confirmed with DNA sequencing. The constructed expression vector was inserted into *E. coli* BL21 (DE3) competent cells for expression. Transformants were selected by plating kanamycin with LB agar.

### 4.2. Expression of the Recombinant hG31P-P-113

1 mL cell culture was inoculated into 100 mL LB medium containing kanamycin (50 μg/mL) and was incubated at 37 °C with 150 rpm shaking for 8 h. 50 mL incubated cell culture was then inoculated into 1000 mL LB broth medium containing kanamycin (50 μg/mL) and was incubated until the absorption OD_600_ reached 0.8. 1 mL of IPTG (100 mg/mL) was added into the medium, and continued to incubate for 6 h. The sample was collected and then centrifuged at 4570 g for 20 min at 4 °C. The supernatant was disposed and the pellets were stocked at −20 °C.

### 4.3. Lysis of Cells and Purification of Recombinant hG31P-P-113

Cell pastes obtained from 1 liter of culture were suspended in Tris buffer (80 mL) with high salt concentration (50 mM Tris, 1 mM EDTA, 700 mM NaCl, 1 mM PMSF, pH 8.0). 0.4mL TritonX-100 (Sigma-Aldrich, St. Louis, MO, USA) was then added. A High-Pressure Homogenizer (AVESTIN EmulsiFlex C3, Mannheim, Germany) was used to lyse the suspended cells. The cell lysates were heated up in 80 °C water for 10 min, and were then immediately cooled down in 0 °C ice water for 10 min. The result was verified with SDS PAGE. The produced proteins were mixed with sample buffer (0.2 M Tris-HCl, pH 6.8, 30% glycerol, 10% sodium dodecyl sulfate (SDS), 10 mM DTT, 0.05% bromophenolblue). The samples were loaded to 12% (wt/vol) SDS-polyacrylamide gel electrophoresis (SDS-PAGE) and then ran the gel at 120 volts for 80 min. Protein bands were detected by Coomassie Brilliant Blue G-250 (J.T. Baker Chemical Co., Phillipsburg, NJ, USA) staining. The supernatant was gathered after centrifugation at 14,800× *g* for 30 min at 4 °C, and followed by dialysis using the cellulose tubular membrane (Cellu·Sep T1 Membrane, Membrane Filtration Products, Inc., Seguin, TX, USA) within 20 mM citrate buffer (pH 6.0) at 4 °C for 6 h.

### 4.4. Cleavage of hG31P-P-113

After dialysis, the protein solution was centrifuged at 14,800 *g* for 30 min at 4 °C. The pellet was suspended and cleaved incubating 16 mL of a cyanogen bromide (CNBr) solution (12.5 mg CNBr/mL, 70% (*v*/*v*) formic acid) in dark for 6 h at room temperature. The solution was then diluted (1:10) with 144 mL of water and dried under vacuum.

### 4.5. Purification of P-113 by HPLC

Following cleavage, all of the samples were purified by using C18 reversed-phase high-performance liquid chromatography (RP-HPLC) on the Waters® Breeze™ 2 HPLC system (Waters, Milford, MA, USA) and using a Reprosil 100 C18 (Dr. Maisch-GmbHc, Ammerbuch, Germany) Semi-preparative column. The column was equilibrated with 10% (*v*/*v*) acetonitrile containing 0.1% (*v*/*v*) trifluoroacetic acid (TFA) and eluted with a gradient step from 10 to 40% (*v*/*v*) acetonitrile for 60 min at a flow rate of 1 mL/min. Signals were detected by UV 220 nm. Each peak was collected and lyophilized. The molecular mass of P-113 was verified by MALDI-TOF mass spectrometry.

### 4.6. Antimicrobial Activity Assays

Strains ATCC 6258, ATCC90028, ATCC13803, YH05001, YH050092, YH050105, YH050075, YH050007, YH050013, and YH0014 were derived from Hsiu-Jung Lo, National Health Research Institute, Miaoli, Taiwan and were published previously [31]. Back to then, there was no need for an IRB or ethical code to obtain the strains from HIV patients. To determine minimum inhibitory concentration (MIC) values of the purified P-113 peptides, the broth microdilution assay was used. Fungus strains were grown overnight in SDB media at 30 °C. These cell cultures were then used to inoculate fresh media and were grown for several hours and subsequently diluted to a final concentration of 5 × 10^5^ CFU/mL (*C. albicans*, OD_600_ = 1 = 10^7^ CFU/mL) in LYM broth. 100 μL of each diluted microbes was transferred into each well of a 96-well plate, into which 1 μL dilution series of P113 peptides in sterile water (ranging from 100 to 0.78 μg/mL) has previously been added. After incubation for 17 to 24 h at 30 °C, the MIC value of purified P-113 was determined to be the lowest concentration that showed no change in optical density.

### 4.7. Circular Dichroism Spectroscopy (CD)

All of the CD measurements were recorded in far UV (195 to 260 nm) by 0.1 cm path-length cell on an AVIV CD spectrometer (Aviv Biomedical Inc., Lakewood, NJ, USA) with programmable temperature controller. The peptide concentration for CD was 60 μM in 20 mM sodium phosphate buffer at pH 7.4.

### 4.8. Nuclear Magnetic Resonance Spectroscopy (NMR)

The NMR experiments were performed on ^15^N labeled samples. The NMR protein samples were prepared in NMR buffer (20 mM sodium phosphate buffer, 10 mM NaN_3_, pH 5.2) with 200 mM perdeuterated d_25_-SDS and 10% D_2_O (*v*/*v*). All of the NMR experiments were recorded at 313 K on a Bruker Avance 600-MHz NMR spectrometer (Bruker, Billerica, MA, USA). Analysis of the spectra was accomplished by using the program Sparky (T.D. Goddard and D.G. Kneller, SPARKY3, University of California, San Francisco, CA, USA).

## Figures and Tables

**Table 1:** Minimum inhibitory concentration (MIC) values of P-113 for tested *Candida* strains (μg/mL).

*Candida* sp.	Source	Strain *	Purified P-113	Synthetic P-113 *
*C. krusei*	ATCC 6258	YLO6	6.25	1.56
*C. albicans*	ATCC 90028	YLO12	6.25	1.56
*C. tropicalis*	ATCC 13803	YLO86	3.13	0.78
*C. albicans*	HIV patient	YH050001	6.25	1.56
*C. dubliniensis*	HIV patient	YH050092	6.25	1.56
*C. glabrata*	HIV patient	YH050105	12.5	3.13
*C. krusei*	HIV patient	YH050075	6.25	1.56
*C. tropicalis*	HIV patient	YH050007	3.13	1.56
*C. tropicalis*	HIV patient	YH050013	3.13	0.78
*C. tropicalis*	HIV patient	YH050114	3.13	1.56

* The less effective antimicrobial activities of the expressed P-113 compared to the chemically synthesized P-113 are due to the amidation of the chemically synthesized peptide [25]. * Strains YH05001, YH050092, YH050105, YH050075, YH050007, YH050013, YH0014 were derived from Hsiu-Jung Lo, National Health Research Institute, Miaoli, Taiwan.
